# Infectious, Contagious and Pestilential Diseases

**Published:** 1867-06

**Authors:** 


### Infectious, Contagious and Pestilential Diseases.

Dr. E. B. Dalton, Sanitary Superintendent Metropolitan Sanitary District, has notified every physician in the Metropolitan Sanitary District to report to the Metropolitan Board of Health all cases under their care of such diseases as have been declared by said Board to be of an infectious, congatious or pestilential character, and that the following have been so declared, viz: cholera, yellow fever, small-pox, ship or typhus, typhoid and scarlet fevers, and measles. It is not intended to make these reports public, or to annoy patients or their families with visits from Sanitary Inspectors, unless when the physician's report shall show necessity therefor. The object is to have a record in this office from which, at any time, physicians or others may derive information as to the prevalence of said diseases and the comparative salubrity in this particular of different sections of the city, and generally of the Metropolitan Sanitary District.

